# Statins alone or polypill for primary prevention of cardiovascular diseases

**DOI:** 10.1186/s40200-016-0280-4

**Published:** 2016-12-07

**Authors:** Shirin Hasani-Ranjbar, Hanieh-Sadat Ejtahed

**Affiliations:** 1 Obesity and Eating Habits Research Center, Endocrinology and Metabolism Molecular-Cellular Sciences Institute, Tehran University of Medical Sciences, Tehran, Iran; 2 Endocrinology and Metabolism Research Center, Endocrinology and Metabolism Clinical Sciences Institute, Tehran University of Medical Sciences, Tehran, Iran

**Keywords:** Polypill, Primary prevention, Cardiovascular disease, Statin

Cardiovascular disease (CVD), the leading cause of death and disability worldwide, imposes huge healthcare costs to society and significant burdens to patients. It is estimated that 18 million deaths occurred from CVD annually worldwide [1]. Over three quarters of CVD deaths take place in low- and middle-income countries including Iran. Therefore, developing and implementing public health strategies is necessary for primary and secondary prevention of CVD [2]. Many of these deaths may be prevented with use of approved medications. Guidelines strongly recommend the use of medicines including aspirin, statins, beta blockers, and angiotensin-converting enzyme (ACE) inhibitors or angiotensin-II receptors blockers (ARBs) for prevention of CVD risk factors including hypertension, dyslipidemia and thrombosis [3]. Nowadays fixed-dose combination therapy is recommended. However, the possible superior clinical effect of polypill compared to statins alone for primary prevention of CVD in intermediate risk population has not been investigated yet.

According to the importance of dyslipidemia, current guidelines recommend statins in the primary prevention of CVD based on predicted cardiovascular risk approach. The American College of Cardiology and American Heart Association (ACC/AHA) statin guidelines recommend high and moderate-high intensity statin therapy for all adults with low density lipoprotein-cholesterol (LDL-C) equal or greater than 190 mg/dl and risk of CVD ≥7.5% over 10 years, respectively [4]. Moreover, ACC/AHA recommends moderate-intensity therapy for adults without diabetes mellitus, aged 40–75 years with 5–7.5% risk of CVD over 10 years [5]. In this regard, Khalili et al. showed that the 2013 ACC/AHA guideline on statin therapy could be useful for both moderate and intensive treatment of hypercholesterolemia and prevention of CVD events in Iranian population [6]. However, Thanassoulis et al. (2016) revealed that an individualized statin benefit approach based on predicted absolute risk reduction over 10 years in comparison with the 10-year risk-based approach could better identify eligible lower-risk individuals who meaningfully benefit from statin treatment [7]. In spite of probable statins adverse effects like muscle pain and increasing serum blood sugar, meta-analysis of randomized trials which evaluated the effect of statin on primary prevention of CVD documented that 1 mmol per liter reduction of LDL-C with statin was associated with 25% lower risk of CVD events [8].

Yusuf et al. (2016) recently published the primary results of the Heart Outcomes Prevention Evaluation (HOPE)-3 study, a multicenter, international, double-blind, randomized, placebo-controlled trial [9–11]. This study was conducted on 12705 intermediate-risk person of various ethnic backgrounds on six continents and 21 countries who did not have CVD with no specific lipid or blood pressure levels for entry. Participants were randomly assigned to rosuvastatin group at a dose of 10 mg per day or placebo and were also randomly assigned to candesartan (16 mg per day) plus hydrochlorothiazide (12.5 mg per day) or placebo for a median follow-up of 5.6 years. They revealed that treatment with rosuvastatin resulted in a 24% lower risk of cardiovascular events compared to placebo. However, blood-pressure lowering treatment did not significantly reduce the risk of cardiovascular events in this intermediate-risk population without cardiovascular disease. Moreover, the comparison of the combined intervention effects with placebo showed no more benefit than rosuvastatin alone [9–11]. The results of HOPE-3 study provide the evidence in support of statin use for primary prevention of CVD. Moreover, Karmali et al. (2016) searched the systematic reviews which evaluated the effect of aspirin, blood pressure-lowering therapy, or statins on cardiovascular events in individuals without prevalent CVD. They showed that aspirin, statins and BP-lowering therapy individually reduced the risk for CVD compared with placebo by 10%, 25% and 16% respectively [3].

Low adherence to prescribed treatments is a major barrier in prevention of CVD. Poor long-term adherence to prescribed medications could be related to social, cultural, psychological and economic factors. Moreover, low availability and affordability of medicines are associated with low adherence. Reducing the number of medications is an approach to increase patient adherence which may be achieved by fixed-dose combination therapy such as polypill. Most of the tested polypills include a statin, an aspirin, a beta blocker, a diuretic or a calcium channel blocker, an ACE inhibitor or an ARB. Chrysant and Chrysant (2016) in five studies including 1142 high-risk participants showed that polypill was usefull for the primary prevention of CVD by decreasing blood pressure and LDL-C concentration [12]. Sepanlou et al. (2012) conducted a meta-analysis to estimate the effect of polypill on primary prevention of ischemic heart disease and stroke in Iranians aged 55 years or older. They revealed that using polypill may reduce ischemic heart disease and stroke deaths by 30% and 53%, respectively [13].

Although a randomized controlled trial is conducting to evaluate the effect of polypill on prevention of CVD in Iranian participants, now there is no evidence of the efficacy, safety and cost-effectiveness of polypill approach for primary prevention of CVD in Iran. Therefore, it seems that health providers should not hurry in polypill recommendation in public health approach. The cost-effectiveness, risk-assessment and acceptability of polypill treatment remain to be investigated. Conducting a randomized clinical trial is recommended to compare the effects of statins with polypill on primary prevention of CVD in Iranian population. Given that generic statins are widely available and affordable in Iran, it seems that it could be an appropriate candidate for prescribing in intermediate-risk population for primary prevention of CVD.

In conclusion, it seems that only patients receiving several drugs with a history of poor adherence to treatments could be good candidates for administration of fixed dose polypill. In other cases, regarding the cost, efficacy and availability of statins, the moderate-intensity therapy of this medication could be applied in primary care in intermediate-risk individuals. Totally, for countries that polypill development and preparation is not cost-effective, it seems that statins alone is a good choice for primary prevention of CVD.

## Abbreviations

ACC/AHA: American College of cardiology and American heart association
ACE: Angiotensin-converting enzyme
ARBs: Angiotensin-II receptors blockers
CVD: Cardiovascular disease
HOPE Study: Heart outcomes prevention evaluation Study
LDL-C: Low density lipoprotein-cholesterol
